# Total and functional parasite specific IgE responses in *Plasmodium falciparum*-infected patients exhibiting different clinical status

**DOI:** 10.1186/1475-2875-6-1

**Published:** 2007-01-04

**Authors:** Joana Duarte, Prakash Deshpande, Vincent Guiyedi, Salah Mécheri, Constantin Fesel, Pierre-André Cazenave, Gyan C Mishra, Maryvonne Kombila, Sylviane Pied

**Affiliations:** 1 Instituto Gulbenkian de Ciencia, LEA CNRS-IGC, Oeiras, Portugal; 2 National Centre for Cell Sciences, Pune, India; 3 Unité d'Immunophysiopathologie Infectieuse, Institut Pasteur, 25 rue du Docteur Roux, 75 724, Paris Cedex 15, France; 4 Département de Parasitologie-Mycologie-Médecine Tropicale, Faculté de Médecine de Libreville, Gabon; 5 Unité des Réponses Immunes Précoces aux Parasites, Institut Pasteur Paris, France

## Abstract

**Background:**

There is an increase of serum levels of IgE during *Plasmodium falciparum *infections in individuals living in endemic areas. These IgEs either protect against malaria or increase malaria pathogenesis. To get an insight into the exact role played by IgE in the outcome of *P. falciparum* infection, total IgE levels and functional anti-parasite IgE response were studied in children and adults, from two different endemic areas Gabon and India, exhibiting either uncomplicated malaria, severe non cerebral malaria or cerebral malaria, in comparison with control individuals.

**Methodology and results:**

Blood samples were collected from controls and *P. falciparum*-infected patients before treatment on the day of hospitalization (day 0) in India and, in addition, on days 7 and 30 after treatment in Gabon. Total IgE levels were determined by ELISA and functional *P. falciparum*-specific IgE were estimated using a mast cell line RBL-2H3 transfected with a human Fcε RI α-chain that triggers degranulation upon human IgE cross-linking. Mann Whitney and Kruskall Wallis tests were used to compare groups and the Spearman test was used for correlations.

Total IgE levels were confirmed to increase upon infection and differ with level of transmission and age but were not directly related to the disease phenotype. All studied groups exhibited functional parasite-specific IgEs able to induce mast cell degranulation *in vitro *in the presence of *P. falciparum *antigens. Plasma IgE levels correlated with those of IL-10 in uncomplicated malaria patients from Gabon. In Indian patients, plasma IFN-γ , TNF and IL-10 levels were significantly correlated with IgE concentrations in all groups.

**Conclusion:**

Circulating levels of total IgE do not appear to correlate with protection or pathology, or with anti-inflammatory cytokine pattern bias during malaria. On the contrary, the *P. falciparum*-specific IgE response seems to contribute to the control of parasites, since functional activity was higher in asymptomatic and uncomplicated malaria patients than in severe or cerebral malaria groups.

## Background

Malaria is a complex disease that kills between one and two million people every year. Most of those affected are children under five years of age, non-immune individuals and pregnant women [1]. The principal cause of death is infection by *Plasmodium falciparum *due to its ability to induce severe complications such as severe anaemia and/or cerebral malaria (CM) often associated with hypoglycaemia [2-4]. The physiopathology of malaria cannot be represented by a single scheme. For example, patients who develop CM present a range of acute neurological manifestations and the disease is characterized by a diffuse encephalopathy, altered levels of consciousness, deep coma and seizure leading to death. Even though during the last few years a lot of information has become available from clinical and experimental studies, the causes of CM remain to be determined. The clinical outcome of a *P. falciparum *infection depends on the genetic factors of the host and parasite, and also on host immune responses. Antibodies and T cells are among the immune factors thought to play a role in mediating protection and also pathology [2-5].

*P. falciparum *infection increases the serum levels of IgM and IgG antibodies but also IgE in individuals living in endemic areas [6-12]. IgEs may protect against or participate in malaria pathogenesis. The association of high anti-*P. falciparum *IgE levels with a reduced risk of developing clinical malaria suggests the involvement of IgE in protection [13,14]. The observation that circulating levels of IgE most often correlate with severe rather than uncomplicated disease suggests a pathogenic role of IgE [8,10-12], and the positive correlation between the levels of IgE/IgE immune complexes and the levels of TNF in CM patients provides supporting evidence [8,10-12]. The exact role played by IgE in malaria is still unclear.

IgE is an immunoglobulin isotype that only exists in mammals. It is present at very low concentrations in the serum of normal individuals, at levels ranging from 10 to 300 ng/ml [9]. Its functional effect has been shown to depends on Fc receptors expressed on mast cells and basophils both in mice and humans, as well as on eosinophils, monocytes/macrophages and platelets in humans [9]. IgEs positively regulate both of their receptors: the high affinity receptor (Fcε RI) and the low affinity receptor (Fcε RII or CD23) [15]. The Fcε RI is expressed only on mast cells and/or basophils in both mice and humans [9,16]. The binding of IgE to the high affinity receptor on the mast cell membrane and its subsequent aggregation with antigens results in degranulation and the release of mediators that further aggravate an ongoing allergic process [17]. On basophils, the cross-linking of Fcε RI-bound IgE rapidly induces the release of IL-4 and IL-13 [16], among other inflammatory mediators. The low affinity receptor (Fcε RII) is the second major and widely distributed IgE receptor. It is also known as CD23 and is constitutively expressed on B cells and is induced by IL-4 on macrophages, some T cells, human eosinophils and platelets [9,16]. The cross-linking of CD23 on macrophages or on other CD23-bearing effector cells by IgE-containing immune complexes is thought to play a pathogenic role in malaria via TNF-mediated pathways [16].

This study aimed to evaluate the total and functional *P. falciparum*-specific IgE responses, the association of these responses with plasma cytokine patterns and the phenotype of the disease in endemic controls and infected patients with different clinical forms of malaria. The infected patients originated from a low endemic area in India and a high endemic area in Gabon.

## Materials and methods

### Study population

#### Patients from Gabon

All the patients included in this study were children aged between 0.1 and 6 years (mean age = 2.6 years) recruited between 1996 and 2000 at the Owendo Pediatric Hospital (OPH) and the Libreville Hospital Center (LHC) in Gabon (see Table 1). Informed parental consent had been obtained. Gabon has an equatorial climate that is hot and humid, with an endemic malaria transmission. The study design was approved by the local health office ethics committee. The patients were distributed into different groups according to World Health Organization (WHO) guidelines for the definition of uncomplicated and severe malaria [18]. A cohort of 135 *P. falciparum*-infected children was constituted and divided into three groups according to disease severity [[6], 67] 50patients with uncomplicated malaria (UM), 29 with severe non-cerebral malaria (SM) developing severe anaemia (haemoglobin level < 5 g/dl), or hypoglycaemia (glycaemia < 2.2 mmol/ml), and 17 with severe cerebral malaria (CM) with a Blantyre Coma Score < 2, or three convulsive episodes during 24 hours before admission with post-critical comatose > 15 minutes]. Two control groups were recruited: an uninfected group, also called endemic control (EC) group, comprising 17 children with *P. falciparum*-negative thin blood smear, and asymptomatic infected group (AI) comprising 22 children with no clinical manifestation of malaria but a *P. falciparum*-positive thin blood smear.

**Table 1:** Characteristics of both studied cohorts: Gabon and India. Clinical group description, according to the number of patients, age, sex and parasitaemia.

	**Clinical Groups**	**Staff**	**Age-mean **(min-max)	**Sex **(male-female)	**Parasitemia **(%)
**Gabon**	**EC**	17	2,7 (0,5–5)	10-7	-
	**AI**	22	2,9 (0,1–5)	12-10	0.22 (0.01–1.2)
	**UM**	50	3 (0,5–5)	22–28	6.88 (0.05–48.6)
	**SM**	29	1,8 (0,2–4,5)	18-11	5.91 (0.08–31)
	**CM**	17	2,4 (0,5–5)	12-5	10.42 (0.15–64)
	**Total**	145	2,6 (0,1–5)	74-61	

**Índia**	**NEC**	9	32,7 (25–63)	8-1	-
	**EC**	14	27,3 (23–37)	13-1	-
	**UM**	31	30,8 (4–70)	18-13	1,24 (0,71–2,18)
	**SM**	13	30,4 (8–65)	10-3	1,11 (0,38–3,21)
	**CM**	26	40,4 (9–72)	16-10	2,04 (1,07–3,87)
	**ExCM**	5	19,36 (7–51)	0–5	-
	**Total**	93	32,3 (4–72)	65-28	1,50 (1,02–2,20)

NEC- non endemic control, EC- endemic control, AI- asymptomatic infected, UM – uncomplicated malaria, SM – severe malaria (non-cerebral), CM – cerebral malaria, ExCM – Ex-cerebral malaria. Note: sex couldn't be determined for 1/18, 11/61 and 4/33 patients from EC, UM and SM, respectively.

#### Patients from India

Malaria patients were recruited in the village of Gondia, an endemic region in the north-east of the Maharashtra State of India. The village is surrounded by forest. Gondia has been known as an endemic area for at least the last 20 years. *P. falciparum *appeared in Gondia over the last 10 years. It is transmitted during the rainy season in June, peaks in the winter season (November, December and January) and becomes rare as summer approaches (March, April and May). The studied groups consisted of 98 patients from four to 72 years of age, being predominantly adults (see Table 1). Six cohorts were constituted according to WHO criteria for uncomplicated and severe malaria: two control groups of uninfected individuals from non-endemic (NEC) and endemic regions (EC) comprising nine and 14 patients respectively; three groups of infected patients, with 31 developing uncomplicated malaria (UM), 13 developing severe non-cerebral malaria (SM) and 26 developing cerebral malaria (CM); and one group of five patients that had recovered from CM (ex-cerebral malaria patients, Ex-CM). Between eight and 10% of the CM malaria cases died. UM cases were treated as outpatients. SM patients were admitted to hospital fully conscious and could respond well verbally to doctors' questions. CM cases were in coma. Drug treatment was paracetamol, quinine and arteether (E-mal^®^). Samples were collected after obtaining the consent of the patients, or of their families. Blood samples from endemic controls were collected from the relatives of malaria patients (brothers/sisters/parents) with their consent. These controls had not suffered from malaria during the previous two years. Non-endemic blood samples were collected from individuals who had not suffered from malaria during the previous five years.

### Blood sample collection and parasite assessment

Venous blood was collected on EDTA in sterile vacutainers from each patient on the day of hospitalization (day 0, before any treatment), and seven (day 7) and thirty days later (day 30). Plasma was obtained by centrifuging the blood samples at 5000 rpm for 15 min. Plasma samples were stored at -80°C until use.

Parasitaemia was assessed by counting asexual forms of *P. falciparum *on thin blood smears under a light microscope after Giemsa staining. Parasitaemia was expressed as the mean percentage of infected red blood cells.

### Culture of malaria parasites

Erythrocytic stages of the *P. falciparum *malaria parasite line *FAN 5HS *(source: NCCS, Pune, India) and 3D7 were cultured using candle jar dessicators as previously described [19]. The culture medium was RPMI 1640 (Gibco-BRL), supplemented with 0.5% AlbuMix (Gibco BRL). The cultures were maintained in six-well or 24-well tissue culture plates (NUNC). Parasitaemia was 5% at the start of culture and reached 25% after six days. Culture medium and fresh RBCs were added every other day.

### Preparation of parasite extracts

Parasite soluble antigen was prepared from synchronous cultures containing more than 20% mature trophozoites; more than 6% rings and more than 5% schizonts were used. The cultures were pooled and centrifuged at 3,000 rpm at 4°C, and the pRBC pellet was kept and the supernatant discarded. The pRBC pellet was suspended in 10 ml sterile PBS 1 × (0.15 M, pH7.2) and then centrifuged. The parasitized red blood cell (pRBC) pellet was washed five times and then lysed by adding 15 ml of 0.1% saponin. The saponin treatment frees the parasites from the infected RBCs. This was centrifuged at 6,000 rpm for 30 min at 4°C. The supernatant was discarded and the parasite pellet was washed five or six times with sterile cold PBS. The parasite pellet was resuspended in 1 ml protein isolation buffer containing a cocktail of protease inhibitors. This was briefly sonicated and the tube was kept at 4°C for between four and five hours. The contents of the tube were agitated by cyclo-mixing and then centrifuged at 6,000 rpm for 30 min at 4°C. The clean supernatant was collected in a separate tube and the pellet was discarded. The contents were sterilized by passing through 0.22 μm-pore filters. Aliquots of the antigen were frozen at -70°C until use. Parasite proteins were quantified by the Bradford method. The concentration of the parasite line FAN 5HS and 3D7 were 1.2 and 2.6 mg/ml, respectively.

### Normal RBC extracts

Normal red blood cell (RBC) extract was prepared from the same batch of RBCs used for culturing the parasites, and followed the same procedure as previously described for pRBCs. Briefly, the RBCs were washed with PBS and the buffy coat was removed. After centrifugation, the RBC pellet was suspended in 1 ml protein isolation buffer containing a cocktail of protease inhibitors. This was briefly sonicated and the tube was kept at 4°C for between four and five hours. The contents of the tube were agitated by cyclo-mixing and then centrifuged at 6000 rpm for 30 min at 4°C. The clean supernatant was collected in a separate tube and the pellet discarded. The protein contents were estimated using a protein determination kit (BCATM protein assay Kit, Pierce, France).

### Total IgE levels

An ELISA method was used to detect total IgE plasma levels in samples corresponding to day 0, day 7 and day 30. ELISA plates (96 microwell plates, reacti-bind 96 EIA Plate 100/PKG, Pierce) were coated with 50 μl/well of purified sheep polyclonal anti-human IgE solution at 5 μg/ml (The Binding Site, Birmingham UK) by incubation overnight at 4°C. The plasma samples were diluted 1:5 and incubated for two hours at 37°C. Bound IgE was detected using a peroxydase-conjugated polyclonal anti-human-IgE (The Binding Site, Birmingham UK). Binding was revealed using the OPD substrate (Sigma) and the product was quantified from the optical density (OD) at 450 nm. Serial dilutions ranging from 2 μg/ml to 0.0019 μg/ml of IgE solution (human monoclonal IgE provided by Dr Thierry Batard – Stallergenes, Anthony, France) gave the standard curve. The median of each optical density value was fitted into the sigmoidal standard curve using a specific ELISA programme running in Igor version 3.16 (Wavemetrics, Lake Oswego, OR).

### IgE functional assay

A new rat mast cell line RBL-2H3 transfected with a human Fcε RI α-chain that triggers degranulation upon human IgE cross-linking was used [20]. Cells were maintained in Dulbecco medium (Gibco BRL, Eragny, France) containing 10% foetal bovine serum (FCS), 100 U/ml penicillin and 100 U/ml streptomycin (GIBCO BRL, France). Cells were expanded by incubation at 37°C for three to four days in complete Dulbecco medium supplemented with G418 (GIBCO BRL, France).

β-Hexosaminidase is known as a component of the basophil and the mast cell specific granule, and is released during degranulation of these cells [21]. Degranulation was monitored after antigen stimulation by measuring the level of released β-hexosaminidase. Fcε RI α-chain RBL-2H3 transfected rat mast cell line cultures (5 × 10^5 ^cells per well) were incubated with the different serum samples at a non-cytotoxic dilution (previously determined) for 48 hours at 37°C in the absence of the G418 antibiotic. The upregulated receptors were saturated by incubation at 4°C for 30 minutes with the same samples diluted 1:10. The cells were then washed with PBS 1X, centrifuged and resuspended in 1 ml Tyrode buffer before being centrifuged again. Finally, the cell pellet was resuspended in 450 μl of D2O (50%) and Tyrode buffer (50%) solution and each culture sample was distributed to 10 ELISA plate wells. Different controls were carried out for each sample. Control cells on lane 1 and 2 were subjected to Triton disruption (Triton 5%) and represented 100% enzyme release. Cells on lanes 3 and 4 were incubated with 50 μl of complemented Dulbecco medium without serum and represented the background enzyme release. Lanes 5 and 6, 7 and 8, 9 and 10 were incubated with 50 μl of different duplicated concentrations of parasite extract (1,000, 100 and 10 ng/ml) for 30 minutes at 37°C. After centrifugation of each well sample, 50 μl of each supernatant was collected and incubated with 50 μl of PNAG substrate solution for 90 minutes at 37°C. The level of released β-hexosaminidase was estimated from the OD at 405 nm using a spectrophotometer. All results are expressed as the percentage of total β-hexosaminidase in the cells after correcting for spontaneous release in unstimulated cultures, calculated as following: (experimental β-hexosaminidase – background β-hexosaminidase)/(total β-hexosaminidase – background β-hexosaminidase) × 100.

### Flow cytofluorometry analysis

FACS analysis was performed after incubating RBL-2H3-D12.8 cells with several dilutions of serum samples to follow the induction of the high affinity receptor (Fcε RI) expression after stimulation by IgEs in the patient's sera. Cells were incubated for 48 hours at 37°C with the different serum samples optimally diluted to avoid cytotoxicity. A saturation step with the same sera diluted 1:10 was done by incubation at 4°C for 30 minutes. Cells were washed with PBS 1X and incubated with FITC-labelled anti-IgE (Tebu, Le Perray en Yvelines, France) (1/100) for 30 minutes. Cells were washed again, centrifuged, resuspended in PBS 1X and analysed by cytofluorometry using Cellquest software (Beckton Dickinson, USA). 10,000 cells were acquired per tube.

### Cytokine levels

The levels of cytokines in the plasma (IL-4, TNF, INF-γ, and IL-10) were estimated by Opti-ELISA kits (Pharmingen, San Diego, CA.USA) used following the manufacturer's instructions.

### Statistics

Due to a non-normal distribution of the scores in each group, non-parametric tests were performed, using the median to compare the different clinical groups. The Mann Whitney test was used for comparisons between two groups and the Kruskall Wallis test to compare three or more groups. Spearman's correlation was used to check for correlations between parameters. P values less than 0.05 were considered as significant. Chi-squared test was used to compare qualitative variables.

## Results

### Serum total IgE levels in groups of *P. falciparum *infected patients with different clinical phenotypes

**Figure 1:**
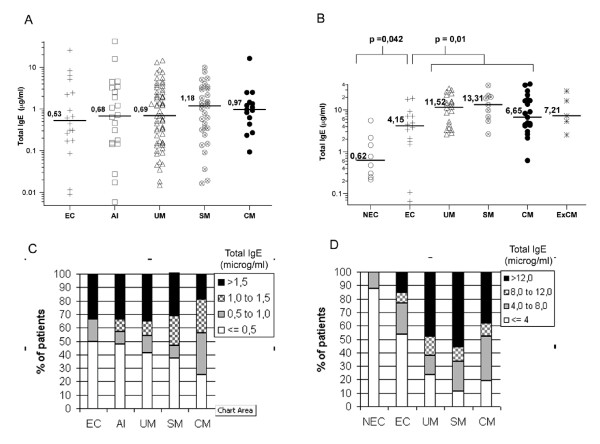
Distribution of total IgE levels per clinical group in both studied populations: Gabon and India. **A. **Total IgE levels (μg/ml) per clinical group in Gabonese patients (non-significant Kruskall Wallis test). **B**. Total IgE levels (μg/ml) in Indian patients (significant Kruskall Wallis test, p = 0.0005). **C. **Percentage of patients with defined IgE levels per group in the Gabonese population (normal levels (N) lower than or equal to 0.500 μg/ml, moderate levels (N to 2N), from 0.501 to 1.000 μg/ml, high levels (2N to 3N), from 1,000 to 1,500 μg/ml, very high (>3N) greater than 1,500 μg/ml). **D. **Percentage of patients with defined levels of IgE per group in the Indian population (normal levels (N) lower than or equal to 4,000 μg/ml, moderate levels (N to 2N), from 4,000 to 8,000 μg/ml, high levels (2N to 3N), from 8,000 to 12,000 μg/ml, very high (>3N), greater than 12,000 μg/ml). Legend: EC – endemic control, AI – Asymptomatic infected, UM – uncomplicated malaria, SM – severe malaria, CM – cerebral malaria, NEC – non-endemic control, ExCM – ex-cerebral malaria.

Total IgE levels were analysed in endemic controls and in cohorts of *P. falciparum*-infected patients with different clinical forms of malaria, ranging from asymptomatic to cerebral disease, from Gabonese and Indian endemic areas to study the association between the IgE response and disease severity. Total IgE levels were measured by ELISA in individual sera before drug administration (corresponding to day 0) and determined the general distribution in the studied populations from Gabon (Figure 1A) and India (Figure 1B). Total IgE concentrations were found to be much higher in patients from India (mainly adults) than in patients from Gabon (children). In both populations independent of the different levels of IgE in each population, the median IgE levels within each clinical group tended to increase upon infection (mainly in UM and SM groups), although the difference between the groups was only significant in the Indian population (Kruskall Wallis, p = 0.0005). As only Indian patients showed a significant difference, the Mann Whitney test was used to compare the different groups in this population only. There was a significant increase in IgE levels in the EC group compared to the NEC group (p = 0.042). The most significant increase in IgE levels (versus the EC group) occurred in the UM patients (p = 0.015) and in the SM patients (p = 0.013). No significant difference between the EC group and the CM and Ex-CM groups was observed.

**Figure 2:**
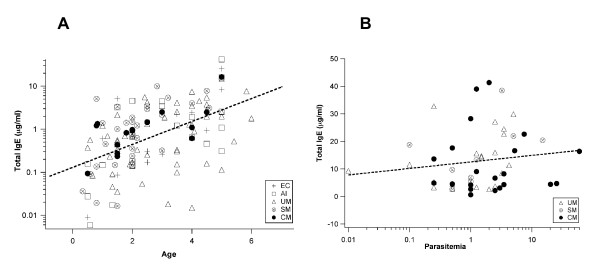
**A. **Total IgE correlation with age in the Gabonese population (significant spearman correlation, p = 1.0 × 10-9). **B. **Total IgE correlation with parasitaemia in the Indian population. Significant Spearman correlation (p = 0,0001).

A range of values of IgE levels was defined enabling the analysis of the frequency of normal, moderate and high IgE levels in each clinical group of patients. The so-called normal values were adjusted to the studied population because the Gabonese and Indian groups had different plasma total IgE ranges. Therefore, the normal value (N) was defined by the median IgE levels in the endemic controls of each study population. Consequently, all values between N and 2N were considered as low/moderate IgE levels and those between 2N and 3N as moderate/high IgE levels, with the highest levels being above 3N (Figures 1C and 1D). Even in Gabonese patients, for whom the increase of IgE in the disease groups was not significant, a higher percentage of patients with clinical disease had higher IgE levels than controls and asymptomatic patients. These differences were more marked in the UM, SM and CM Indian patients (Figure 1D). In the Indian population, the NEC group did not have moderate/high IgE levels, although a high percentage of patients exhibited normal IgE levels (Figure 1D). Also, no significant change was detected in IgE levels over time in the UM, SM and CM groups of the Gabonese cohorts when tested seven days and 30 days after treatment (Table 2). No significant association of malaria and IgE levels with sex in the two studied populations. However, a significant increase in IgE levels with age (p = 0.00034) was observed in the Gabonese subjects (Figure 2A) but not in the Indian subjects.

**Table 2:** Day 0, day 7 and day 30 median IgE levels per clinical group in the Gabonese population.

**Total IgE (μg/ml)**	**EC (min-max)**	**AI (min-max)**	**UM (min-max)**	**SM (min-max)**	**CM (min-max)**
**Day 0**	0,525 (0,009–25,47)	0,677 (0–41,96)	0,690 (0,015–13,173)	1,132 (0,016–9,915)	0,922 (0,093–16,34)
**Day 7**	0,582 (0,0144–5,644)	0,448 (0,0192–3,188)	0,516 (0,011–5,228)	0,836 (0,016–5,689)	8,033 (8,033–8,033)
**Day 30**	1,026 (0,044–2,628)	0,977 (0,056–7,446)	0,151 (0,052–3,925)	0,662 (0,011–6,315)	------

EC – endemic control, AI – asymptomatic infected, UM – uncomplicated malaria, SM – severe malaria (non-cerebral), CM – cerebral malaria.

The correlation between IgE levels and the parasite load was tested. Although the general trend was different in the Indian and Gabonese population, there was no significant correlation between IgE concentration and parasite load for all groups together. In Gabonese cohorts, a negative correlation for all groups was observed, except for the UM patients where the correlation showed a positive tendency. In the Indian cohorts, a positive correlation was observed between IgE levels and parasite load, mainly in the UM and SM groups (Figure 2B).

### Functional parasite specific IgE response in *P. falciparum *infected patients

**Figure 3:**
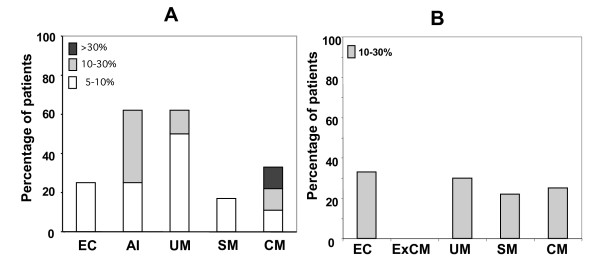
Percentage of patients with positive functional IgE against parasite antigen in the Gabonese and Indian populations. **A. **Distribution of patients with positive anti-parasite functional IgE, exhibiting different intensities of enzyme release per clinical group in the Gabonese population (low enzyme release, from 5 to 10%; moderate enzyme release, from 10 to 30%; and high enzyme release, greater than than 30%). **B. **Distribution of patients with positive anti-parasite functional IgE in the Indian population.

Previous studies have used ELISA to quantify specific IgE present in the serum [7,8,10,11]. The functionality of specific IgEs present in the serum was studied by evaluating the ability of these IgEs to induce mast cell degranulation in the presence of the parasite antigen. A rat mast cell line transfected with the human α-chain of Fcε RI was used [20]. Human Fcε RI expression was induced after incubation with all serum samples at non-cytotoxic dilutions. FACS was used to detect the presence of FcåRI receptors on the mast cells surface induced by IgE present in serum samples. Although the fluorescence intensity revealing human Fcε RI expression by the mast cells varied between patient samples, IgE receptors were upregulated in all the samples tested. No correlation between total IgE levels in the serum and the up-regulation of mast cell receptors was found. There was no significant correlation between IgE levels and receptor upregulation and the level of mast cell degranulation. Sensitized cells were then incubated with different concentrations of a *P. falciparum *blood-stage antigen extract. Mast cell degranulation was measured by quantifying the release of β-hexosaminidase. Control cells not exposed to any serum gave a maximum mast cell degranulation of 3%. Therefore, serum samples giving an enzyme release greater than 5% in the presence of at least one of the antigen concentrations were considered positive for functional IgE. In the Gabonese cohorts (Figure 3A), there were functional *P. falciparum *IgE in all clinical groups. However, the percentage of patients with functional specific anti-parasite IgE was higher in asymptomatic and uncomplicated malaria patients than in other groups. Also, the percentage of patients displaying parasite-specific IgE was lower in the group exhibiting severe disease. The distribution of patients per group releasing between 5 and 10%, 10 and 30% and above 30% β-hexosaminidase induced by specific anti-parasite IgE revealed that one patient in CM group had a degranulation level above 30%, being the highest induced response among all the tested individuals. The same assay was carried out on the Indian population. Although there was no significant difference between groups, the percentage of patients having functional IgE recognizing the parasite extract was slightly higher in EC and UM groups than in SM and CM groups (Figure 3B). All positive patients had an enzyme release of between 10 and 30%. No significant correlation was found between *P. falciparum*-specific IgE-induced mast cell degranulation levels and sex, age and parasitaemia.

### Relationship between total and specific serum IgE and cytokine profiles

**Figure 4:**
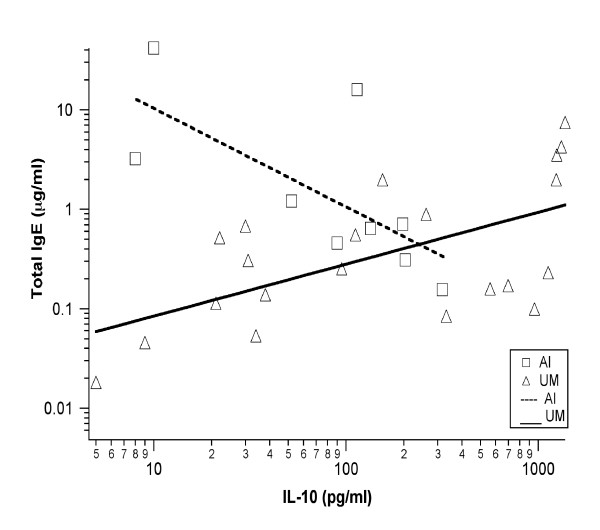
IL-10 correlation with total IgE levels in the Gabonese population. **Dashed line - **Asymptomatic patients (significant negative spearman correlation, p = 0.025). **Bold line -  **Uncomplicated malaria patients (significant positive correlation, p = 0.017).

**Figure 5:**
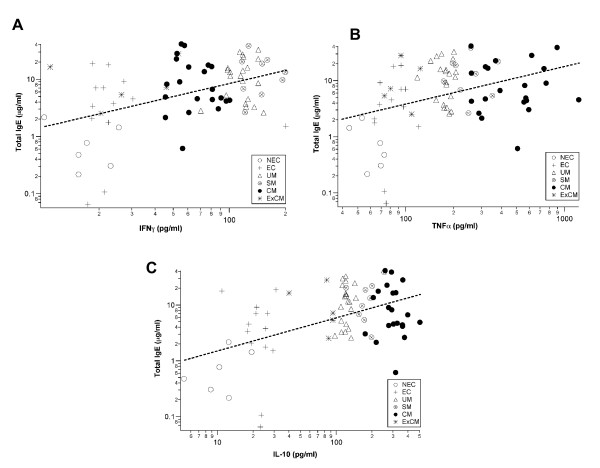
Cytokine correlation with IgE levels in the Indian population. **A. **TNF correlation with total IgE levels (significant positive spearman correlation, p = 0.0037); **B. **IFN-γ correlation with total IgE levels (Significant positive spearman correlation, p = 0.0028); **C**. IL-10 correlation with total IgE levels (significant positive spearman correlation, p = 0.0051).

IgE production is influenced by cytokines produced by activated T cells. These cytokines are also involved in pathophysiological mechanisms associated with severe malaria [4,22,23]. Therefore, the relationship between the cytokine profile, the IgE levels and the clinical manifestation was investigated. IFN-γ, TNF and IL-10 levels were measured in the sera of the Indian and Gabonese patients. IL-4 levels were measured only in Indian patients as it was not different between the Gabonese groups. IFNγ concentrations were highest in the Gabonese AI and CM groups (Table 3). This cytokine is significantly higher in the AI and CM groups than in EC (p = 0.02 and p = 0.009 respectively). The plasma TNF concentration was similar in the severe SM and CM groups and in disease-free EC and AI groups. TNF levels were clearly higher in SM and CM groups than in the UM group (p < 0.001). Surprisingly, EC and AI also exhibited higher TNF levels when compared to UM group. No association between IFN-γ or TNF and IgE levels were found. However, a significant positive correlation was found between the concentration of total IgE and IL-10 in the UM group (p = 0.02) and a significant negative correlation in the AI group (p = 0.02) (Figure 4). The median levels of the different cytokines in the plasma of Indian patients are given in Table 4. IL-10 and TNF levels were higher in CM patients than in controls and other *P. falciparum*-infected patients. The plasma concentrations of these cytokines were moderate in cured CM patients (ExCM). Their levels of IL-10 and TNF were slightly higher in endemic controls than in non-endemic controls. Levels of IFN- were lower in the CM group than in AI group. No difference was found for the levels of IFN-γ between the uncomplicated and severe disease. Although, there was a significant correlation between IgE levels and IFN-γ (Figure 5A), TNF (Figure 5B) and IL-10 (Figure 5C) levels when looking at all the groups combined. Most diseased groups had high cytokine levels, whereas control groups had lower levels (Figure 5A, B and 5C).

**Table 3:** Cytokine distribution in the Gabonese population: median TNF, IFN-γ and IL-10 levels per clinical group.

	**EC (min-max)**	**AI (min-max)**	**UM (min-max)**	**SM (min-max)**	**CM (min-max)**
**TNF (pg/ml)**	41 (8–94)	36 (0–395)	8 (0–440)	175 (1–442)	209 (0–1520)
**IFN-**γ **(pg/ml)**	3,2 (0–8)	9,5 (0–31)	5 (0–395)	4 (0–201)	6,2 (2,5–9)
**IL-10 (pg/ml)**	14 (0–83)	102 (0–317)	134 (0–1380)	339 (0–5200)	95 (0–2300)

EC – endemic control, AI – asymptomatic infected, UM – uncomplicated malaria, SM – severe malaria (non-cerebral), CM – cerebral malaria.

Comparing group by group with the Endemic control: TNF was significantly lower in UM (p = 0,0006); IFN-γ was significantly higher in AI and CM (p = 0,02 and p = 0,007, respectively); IL-10 was significantly higher in UM (p = 0,0009), SM (p = 0,0001) and CM (p = 0,0001)

**Table 4:** Cytokine distribution in the Indian population: median TNF, IFN-γ and IL-10 per clinical group.

	**NEC (min-max)**	**EC (min-max)**	**UM (min-max)**	**SM (min-max)**	**CM (min-max)**	ExCM **(min-max)**
**TNF (pg/ml)**	57 (39–73)	78 (63–120)	180 (119–207)	200 (173–381)	530 (258–1227)	81 (69–124)
**IFN-**γ **(pg/ml)**	23 (10–31)	22 (17–201)	119 (70–153)	127 (111–200)	65 (45–101)	32 (11–53)
**IL-10 (pg/ml)**	13 (5–26)	22 (11–31)	120 (97–147)	176 (121–253)	301 (175–506)	85 (40–108)
**IL-4 (pg/ml)**	18 (8–40)	58 (23–84)	65 (31–204)	69 (50–89)	62 (40–85)	48 (29–80)

NEC – non endemic control, EC – endemic control, UM – uncomplicated malaria, SM – severe malaria (non-cerebral), CM – cerebral malaria, ExCM – Ex-cerebral malaria.

Comparing group by group with the Endemic control: TNF was significantly lower in NEC (p = 0,0006) and higher in UM (p = 1,19 × 10-7), SM (p = 1,01 × 10-5) and CM (p = 2,26 × 10-7); IFN-γ was significantly higher in all diseased groups (UM – p = 5,11 × 10-6, SM – p = 1,01 × 10-6, CM – p = 9,71 × 10-6); IL-10 was significantly higher in all diseased groups (UM – p = 1,04 × 10-7, SM – p = 1,01 × 10-5 and CM – p = 2,46 × 10-7).

## Discussion

The main feature of this study is the comparison of total and functional *P. falciparum*-specific IgE responses in two populations of low and high malaria transmission levels, India and Gabon respectively and their relationship with disease severity. Several clinical groups were compared: endemic non-infected controls, asymptomatics and different clinical manifestations including uncomplicated, severe non-cerebral and cerebral malaria.

Indian and Gabonese individuals exhibited a different range of plasma levels of circulating IgE These cohorts had a different age range, with the Gabonese groups being children from 0 to 6 years of age, whereas Indian patients had a mean age of 30 years. In the two populations, irrespective of its concentration and also consistent with previously published data, the IgE distribution tended to increase upon parasite stimulation [8,10,11]. In the Indian population, circulating IgE levels were seven times higher in endemic controls than in non-endemic controls. This suggests that exposure to the parasite strongly influences the production of IgE, although this difference may also be due to other endemic factors [7]. Nevertheless, it was reported that IgE levels were greatest in patients developing severe disease than in CM group (Figure 1A and 1B). When considering the percentage of patients that produce high levels of IgE per group, it was shown that IgE levels are higher in UM and SM (Figure 1C and 1D) than in CM patients who had values similar to that of the controls [8,24]. In addition, the median levels of circulating IgE in the ExCM group were close to that of the CM group. Also, no significant correlation was found between both IgE and TNF levels in the CM group. These observations are contrary to published data describing increased IgE levels that correlate with high concentrations of circulating TNF, a cytokine associated with malaria severity and also with pRBC adherence on brain capillary endothelial cells [9,10,25,26]. The results suggest that either IgE does not play an important role in CM pathogenesis, or that these antibodies may participate in the parasite sequestration into the brain or other organ capillaries [24].

There was a significant increase in IgE levels with age in Gabonese children independent of the disease group (Figure 2). This increase in IgE production between 0 and six years of age may also reflect an increase in the capacity of the immune system to respond to parasite infections [7,27]. Such a correlation was not found in Indian groups. This would be expected because the Indian groups comprised mainly adults, with the few children being older than five years. Although most of individuals in the Gabonese endemic control group had already been in contact with the parasite, as demonstrated by the high titres of specific antibody to *P. falciparum*-infected red blood cells observed in these children, the median plasma IgE concentrations were compared with those of the Indian NEC group. Although the median total IgE levels in the Indian NEC groups was higher than that of the Gabonese EC group, suggesting that age could be an important factor in IgE production, the higher IgE levels in the Indian population may also be interpreted as the result of either environmental factors, such as predominance of food allergies, of the genetic background, which may predispose to developing IgE responses [13,27,29-32] or of a co-infection with other parasites, such as helminths affecting the IgE responses in these patients [33,34]. In the Gabonese cohorts, IgE levels tended to correlate negatively to parasitaemia except in UM whereas all patient groups from India showed a positive correlation.

The pool of circulating IgE comprises both monomeric and complexed immunoglobulins [35,36]. A functional test was performed, based on the ability of the circulating IgE from the sera of different patient groups to induce degranulation of mast cells in the presence of pRBC antigens to better estimate the *P. falciparum *specific IgE response. This test does not provide specific IgE concentration within total IgE. It is based on the specific IgE induced percentage of mast cells degranulation. Degranulation was measured by quantifying β-hexosaminidase release. Functional *P. falciparum*-specific IgEs were detected in randomly chosen patients from all groups in both the Gabonese and Indian populations, except for ExCM Indian patients. The highest percentage of patients with functional anti-parasite IgEs was found in the Gabonese AI and UM and Indian EC and UM groups, which decreased in the SM and CM groups (Figure 3A and 3B). This suggests a protective role for *P. falciparum*-specific IgE, and is consistent with previous published data [13,14].

Although the CM group had a low percentage of patients able to induce degranulation, it was the only group where there was one patient serum inducing a mast cell degranulation above 30%. This intense response may be associated with the presence of IgE with higher affinity for *P. falciparum *antigens, as previously reported by Gonzalez-Espinosa *et al*. [37]. However, the level of degranulation can also be enhanced by the number of receptors involved in recognizing the antigen-IgE complex, which can strongly affect the size of the secretory response [37,38]. There was no evident correlation between the level of functional *P. falciparum*-specific IgE (percentage of enzyme release) and the level of total IgE per group within each population. This is unsurprising, given that both the monomeric and complexed forms of circulating parasite-specific IgE can affect the level of degranulation [39-41]. It is also acceptable that total IgE levels were not directly correlated to FcåRI upregulation levels [40,42]. The minimum level of receptors occupied by the parasite antigen-specific IgE complex required to induce a degranulation response is 10%. Consequently, this response will be independent of the total IgE levels [20]. An increase in specific IgE levels has been also seen in other parasitic infections, such as helminthiasis, in which the specific IgEs usually help to eliminate the pathogens either through hypersensitivity reactions resulting from mast cell degranulation or by inducing antibody-dependent cell-mediated responses [27].

High levels of IgE in *P. falciparum*-infected individuals have been shown to be due to an underlying imbalance in favour of IL-4 in the ratio of CD4^+ ^T cell producers, which are responsible for the IgG/IgM isotype switching to IgE [10]. High levels of circulating IL-4 have also been associated with a greater parasite antigen-specific production of IgE in individuals less susceptible to malaria [13]. Also, Th1-type pro-inflammatory cytokines, such as IFN-γ and TNF are thought to play an important role in the both protecting against and increasing the pathogenesis of cerebral malaria [3,4,22]. Plasma TNF, IFN-γ and IL-10 levels were measured in both the Indian and Gabonese groups. In the Gabonese groups, no direct correlation was found between IgE and IFN-γ or TNF levels in the UM, SM and CM patients. However, the level of IgE production was correlated to IL-10 levels in UM and AI patients (Figure 4). In this group, there was a positive association between IL-10 levels and parasite load, but not between IgE levels and parasitaemia. In the Indian groups, IFN-γ, TNF and IL-10 levels were all significantly correlated with IgE levels independent of the group (Figures 5A, B and 5C). When looking for a correlation per group, no significant statistic value was found. IL-4 is the main cytokine responsible for IgE production, inducing antibody isotype switching from IgG and IgM to IgE [10]. Previous studies have shown an association between the IL-4/IFN-γ levels and IgE levels, suggesting an induced Th2-type switched response [43]. As IL-4 levels were different between groups only in Indians, a correlation was expected between this ratio and IgE levels, and surprisingly, the results showed an opposite correlation (p = 0,012). The levels of IL-4 remained almost constant across all groups as IgE levels increased. Therefore, this opposite correlation arises due to a parallel increase in both IFN-γ and IgE levels. This suggests that, in the Indian population, IL-4 does not seem to directly influence IgE levels. It has been shown experimentally that there are conditions under which alternative mechanisms may induce IgE production independent of IL-4 [44]. Regarding other cytokine ratios, like TNF/IL-10, no significant correlation was found with total IgE levels.

In conclusion, these results showed that total IgE levels increased in infected patients, mainly in UM and SM patients but not in CM patients. The decrease in total IgE levels in the CM group was associated with a higher intensity of mast cell degranulation induced *in vitro *by *P. falciparum*-specific IgE. In addition, no correlation was found between total and functional *P. falciparum*-specific IgE levels.

The results reported here show the high activity of functional circulating *P. falciparum-*specific IgE in asymptomatic malaria patients. However, no correlation was observed between plasma levels of total IgE, the disease phenotype and the cytokines pattern in the different groups of patients studied. The opposite association between pro- and anti-inflammatory cytokine ratios and IgE levels reveals the complexity of immune response disruption occurring during malaria in patients from low and high malaria endemic region.

## Authors' contributions

Joana Duarte contributed to the acquisition, analysis, interpretation of data and manuscript drafting but not the design of the study. Prakash Deshpande and Vincent Guiyedi have highly contributed to conception and interpretation of data and manuscript revising. Salah Mécheri collaborated to the design and interpretation of IgE functional tests and manuscript drafting. Constantin Fesel contributed to the statistical analysis, interpretation of data and manuscript drafting.

Pierre-André Cazenave, Gyan C. Mishra and Maryvonne Kombila have contributed to conception of the study and manuscript revising. Sylviane Pied participated to conception, design, analysis and interpretation of data, the final approval of the version to be published.

## Financial support

This work was part of the *Centre National de la Recherche Scientifique-Laboratoires Européens Associés "Génétique et developpement de la tolérance naturelle*" program. It was supported by the PAL+ program of the French Ministry of Research. We thank the Indo-French Centre for the Promotion of Advanced Research (IFCPAR), New Delhi, India for providing financial assistance (project No.2103-3). C-F received a post-doctoral fellowship from the *Fundaçâo para a Ciência e Tecnologia *(Portugal). V-G holds a fellowship from the *Agence Universitaire de la Francophonie *(AUF).
